# Biogenic Zinc nanoparticles: green approach to synthesis, characterization, and antimicrobial applications

**DOI:** 10.1186/s12934-025-02788-9

**Published:** 2025-07-18

**Authors:** Myada S. M. Ouf, Mahmoud E. A. Duab, Dina I. Abdel-Meguid, Ebaa E. EL-Sharouny, Nadia A. Soliman

**Affiliations:** 1 Botany and Microbiology Department, Faculty of Science, Alexandria, Egypt; 2 Bioprocess Development Department, Genetic Engineering and Biotechnology Research Institute (GEBRI), City of Scientific Research and Technological Applications (SRTA-City), Universities and Research Institutes Zone, New Borg El-Arab City, Alexandria P.O. 21934 Egypt

**Keywords:** Biosynthesis, *Pseudomonas* sp., *Achromobacter* sp., Microbial proliferation, Sludge remediation

## Abstract

**Background:**

Biogenic synthesis of zinc nanoparticles (ZnNPs) has attracted significant interest due to their unique properties and potential biological applications. Unlike chemical and physical methods, biogenic synthesis offers a greener and more eco-friendly alternative. This study explores the synthesis of zinc-based nanoparticles using two distinct bacterial strains.

**Results:**

In this study, zinc nanoparticles were synthesized in two forms: single-phase zinc sulfide nanoparticles (ZnS NPs) and mixed-phase zinc sulfide-oxide nanoparticles (ZnS-ZnO NPs), using *Achromobacter* sp. S4 and *Pseudomonas* sp. S6. The synthesis conditions were optimized for each strain, with pH playing a crucial role: *Achromobacter* sp. S4 favored basic conditions (pH 8.0) for zinc nanoparticles production, while *Pseudomonas* sp. S6 preferred acidic conditions (pH 4.7). TEM analysis revealed that Zn NPs from *Pseudomonas* sp. S6 were rod-shaped, whereas those from *Achromobacter* sp. S4 were spherical. Further characterization using EDX, XRD, and FTIR confirmed the successful synthesis of single phase ZnS NPs and hybride phase ZnS-ZnO NPs. Antimicrobial dose-response testing showed that single-phase ZnS NPs inhibited *Klebsiella pneumoniae*, while mixed-phase ZnS-ZnO NPs were effective against *Staphylococcus epidermidis* at 100 µg/ml based on inhibition zone measurements.Furthermore, the mixed-phase ZnS-ZnO NPs at 25 µg/ml demonstrated superior inhibition of microbial growth in sludge samples, likely due to a synergistic effect.

**Conclusion:**

The study demonstrates the successful biogenic synthesis of ZnS NPs, and ZnS-ZnO NPs using two bacterial strains, with distinct morphological and functional properties. The use of two bacterial species was to assess strain-specific differences in nanoparticle synthesis and performance. The synthesized nanoparticles exhibited promising antimicrobial and environmental remediation potential, highlighting their applicability in both biomedical and environmental fields.

## Background

Nanotechnology is a transformative field that intersects multiple disciplines, including biology, chemistry, physics, materials science, and medicine. Among its most promising applications is the development of engineered nanomaterials, particularly metal-based nanoparticles, which exhibit unique physicochemical properties distinct from their bulk counterparts. These properties—such as high surface area-to-volume ratio, tunable optical characteristics, and enhanced reactivity—have propelled their use in catalysis, drug delivery, biosensing, imaging, and antimicrobial treatments [1]. Within this domain, zinc-based nanoparticles (ZnNPs) have emerged as particularly versatile due to their broad-spectrum antimicrobial activity, low cytotoxicity, biocompatibility, and environmental safety. Zinc oxide (ZnO) and zinc sulfide (ZnS) nanoparticles are the most widely studied forms.

ZnO nanoparticles are known for their high photocatalytic efficiency and UV-blocking properties, making them suitable for applications in medical devices, sunscreens, and food packaging [2]. They have also demonstrated enhanced antibacterial activity against pathogens such as *Staphylococcus aureus* [3] and are increasingly explored for their microbiostatic effects in biomedical applications [4]. Ammu et al. [5] further contribute to this body of knowledge by demonstrating the biosynthesis of ZnO nanoparticles using *Carica papaya* and *Cymbopogon citratus* leaf extracts. Their comparative study highlights how plant-mediated synthesis can influence nanoparticle morphology and structural features, offering an eco-friendly and sustainable route for nanoparticle production.

Meanwhile, ZnS nanoparticles, with their wide band gap (~ 3.7 eV) and excellent photoluminescence, are important semiconductors used in optical coatings, photoconductors, optoelectronics, and pharmaceutical formulations. Recent findings suggest their growing relevance in electrochemical energy storage applications, such as supercapacitors, owing to favorable structural and electrical properties [6]. These nanoparticles typically present as white to yellowish powders and crystallize in either wurtzite (hexagonal) or zinc blende (cubic) structures. They also exhibit near-complete solubility in water, further increasing their utility in biomedical formulations.

A key advancement in the production of ZnNPs is the shift toward green synthesis, which utilizes biological systems instead of harsh chemical reagents. This method has gained momentum as a sustainable, cost-effective, and environmentally friendly alternative to traditional chemical or physical synthesis. It avoids toxic solvents, extreme conditions, and high energy input while enhancing nanoparticle stability and biological compatibility [7]. In support of this, Cengiz et al. [8] synthesized selenium and zinc oxide nanoparticles using *Rheum ribes* whole plant extract, demonstrating significant anticancer and antimicrobial properties, thereby reinforcing the biomedical relevance of green-synthesized nanomaterials.

A diverse range of biological systems—plants, fungi, algae, yeasts, bacteria, and even viruses—has been employed in the biosynthesis of nanoparticles [7]. In microbial systems, both intracellular and extracellular synthesis mechanisms have been documented. For example, *Lactobacillus* spp. produce ZnO nanoparticles intracellularly [9], whereas *Aspergillus aeneus* and *Pichia fermentans* synthesize ZnO nanoparticles extracellularly [10, 11], respectively. Extending this knowledge, Omran and Baek [12] have demonstrated the dual extracellular biosynthesis of cobalt and zinc oxide nanoparticles using *Aspergillus sojae* mycelial-cell free filtrate, reporting significant antibacterial activity and emphasizing the biotechnological promise of fungal systems. In addition to ZnO, early biosynthesis of ZnS nanoparticles was associated with sulfate-reducing bacteria such as *Desulfovibrio* spp [13]., and more recently with *Thermoanaerobacter* spp [14]. Moon et al. [15] further reported the formation of hexagonal ZnS (10 H and 2 H structures) using *Schizosaccharomyces pombe* and *Candida glabrata*, while *Desulfovibrio desulfuricans* was shown to mediate the formation of inorganic ZnS via biologically produced hydrogen sulfide [16].

More recently [17], reported a novel biosynthetic route involving *Callistemon viminalis* leaf extract phytochemicals to synthesize a silver–ruthenium bimetallic ZnO nanocomposite. Their work is particularly notable for its application in nanocoating and photocatalytic disinfection of *Escherichia coli*, illustrating the expanding scope of green nanotechnology in water purification and antimicrobial technologies. Omran et al. [18] further support this trend by developing biologically inspired nanoformulations to control bacterial canker pathogens such as *Clavibacter michiganensis* subsp. *michiganensis* and *capsici*. Their research exemplifies the agricultural relevance of metal-based nanoparticles and their role in integrated disease management strategies.

Despite substantial progress, biosynthetic methods remain underexplored for many microbial taxa. Comparative evaluations of different strains under controlled biosynthetic conditions are limited, and although ZnO nanoparticles dominate the literature, there is a relative scarcity of studies focusing on ZnS or hybrid ZnS–ZnO nanostructures. Such kind of hybrid materials, as noted by Rahal et al. [19], may exhibit synergistic optical and antimicrobial functionalities, making them highly attractive for diverse biomedical and environmental applications.

Physicochemical characterization is essential to validate the successful synthesis of nanoparticles and assess their quality. Key parameters include particle size, morphology, crystallinity, elemental composition, and surface functional groups. A suite of analytical techniques is typically employed, including UV–Visible spectroscopy, dynamic light scattering (DLS), scanning and transmission electron microscopy (SEM/TEM), energy-dispersive X-ray spectroscopy (EDX), X-ray diffraction (XRD), and Fourier-transform infrared spectroscopy (FT-IR) [20]. Additionally, a visible color change during synthesis—typically from colorless to yellow or white—often serves as a preliminary qualitative indicator of nanoparticle formation due to the reduction of metal ions and nucleation of nanoscale particles [21].

**Fig. 1:**
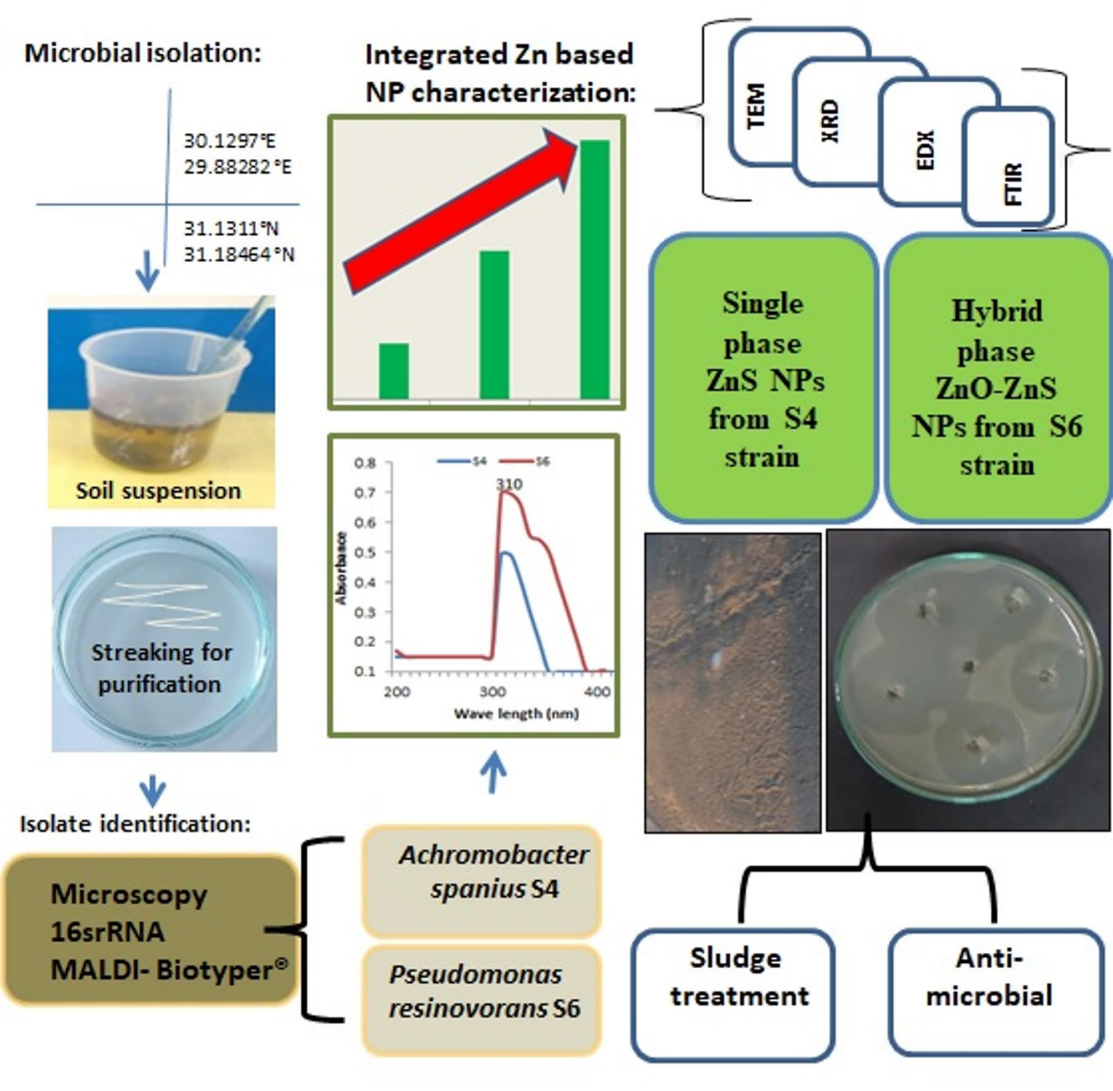
Schematic diagram for the work done in the study

This study aims to: (1) explore the microbial synthesis of zinc nanoparticles using *Achromobacter spanius* S4 and *Pseudomonas resinovorans* S6; (2) characterize the physicochemical properties of the synthesized zinc nanoparticles; and (3) evaluate their antimicrobial activity and potential application in wastewater treatment. A schematic overview of the experimental workflow is presented in Fig. 1, outlining the key steps and methodologies employed throughout the study.

In alignment with the United Nations Sustainable Development Goals (SDGs), this research supports SDG 3 (Good Health and Well-being) through the investigation of antimicrobial properties of biogenic zinc nanoparticles, SDG 6 (Clean Water and Sanitation) by addressing microbial contamination in wastewater, and SDG 12 (Responsible Consumption and Production) by promoting an environmentally friendly, biosynthesis-based approach to nanoparticle production.

## Methods

### Sample collection and isolation

Soil samples were collected from the rhizosphere of mango, peach, and apple plants located along the Mahmoudia Canal, which passes through Kafr El Dawwar in Beheira Governorate and central Alexandria Governorate, Egypt. The sampling sites are approximately located at two coordinates: (31.1311°N, 30.1297°E) and (31.18464°N, 29.88282°E), respectively. One gram of each soil sample was suspended in 10 ml of sterile water, thoroughly shaken, and plated on Luria-Bertani (LB) agar medium containing tryptone, yeast extract, and NaCl at concentrations of 1.0, 0.5, and 0.5%, respectively, along with 1.5% (w/v) agar to isolate bacterial strains. Pure colonies were subcultured and preserved on LB slants at 4 °C. The isolates were then screened for their ability to synthesize zinc nanoparticles (ZnNPs).

### Identification of the selected isolates

#### Transmission electron microscopy (TEM) examination

TEM analysis was performed using a JEOL JEM-1400 Plus electron microscope. TEM was employed to examine the ultrastructure and to measure the size of cells and nanoparticles, providing detailed insights into their morphology. Bacterial cells were prepared for TEM following a standard protocol [22, 23]. Cells were fixed in 2.5% glutaraldehyde and post-fixed in 1% osmium tetroxide, dehydrated through a graded ethanol series, and embedded in LR White resin. Ultrathin sections (~ 80 nm) were stained with uranyl acetate and lead citrate, then examined using a JEOL JEM-1400 Plus electron microscope operating at 120 kV to assess the ultrastructure of the cells.

#### Matrix-assisted laser desorption/ionization MALDI Biotyper^®^

The procedure involves selecting a single microbial colony, suspending it in 70% ethanol for inactivation, and centrifuging to concentrate the cells. After discarding the supernatant, the cells are re-suspended in 70% formic acid to disrupt the cell wall, followed by the addition of acetonitrile for protein extraction. The sample is vortexed and centrifuged again. One microliter of the supernatant is spotted on a target plate, evaporated, and overlaid with a matrix solution for analysis. This method requires an initial concentration of 5 to 10 million cells/ml for effective identification [24, 25] Recent advancements use Teflon-coated stainless- steel plates to improve crystallization and reduce cross-contamination [26].

MALDI spectra typically detect intracellular proteins, mostly in the ranges of 4,000 to 15,000 Da [27]. Lysis of microorganisms with organic solvents in acidic conditions extracts basic cytoplasmic proteins, such as ribosomal, mitochondrial, cold shock, heat shock, DNA binding, and RNA chaperone proteins [28, 29]. These conserved housekeeping proteins are ideal biomarkers for species identification. The MALDI Biotyper^®^ system uses Matrix-Assisted Laser Desorption/Ionization Time-of-Flight Mass Spectrometry (MALDI-TOF MS) and mass spectral pattern matching for the identification of bacterial species from databases [30].

### Molecular characterization

A molecular approach based on partial sequencing of *16 S rRNA* gene was carried out to identify the selected isolates. The genomic DNA was isolated according to Marahatta et al. [31]. Standard protocol for amplification of *16 S rRNA* at annealing temperature (55 °C) was carried out in a thermal cycler (Biorad, USA) [32]. The purified PCR product was subjected to sequencing based on Sanger method [33]. The BLAST program www.ncbi.nlm.gov/blast was used to assess the DNA similarities of the obtained *16 S rRNA* gene sequence. Subsequently, the sequences were deposited in the GenBank for allotment of accession numbers.

### Extracellular synthesis of ZnNPs

Bacterial strains were cultured in LB broth at 37 °C under stirring (120 rpm). After incubation, cultures were centrifuged at 10,000 rpm for 10 min, and the supernatants were tested for ZnNP formation. To screen for ZnNP production, 10 ml of 100 mM ZnSO₄ was added to 10 ml of culture supernatant. Control consisted of ZnSO₄ mixed with sterile medium. The reaction mixtures were incubated for 72 h at 37 °C, 120 rpm [34], and absorbance was measured using UV-Visible spectroscopy (200–800 nm) [35].

### Monitoring the factors affecting ZnNPs synthesis

#### Effect of culture age

Samples were taken every 24 h along 96 h from cultures incubated in LB broth at 37 °C. Cell-free supernatants were mixed with 10 ml ZnSO₄ (100 mM) and incubated for 72 h at 37 ºC, light conditions and stirring (120 rpm). After centrifugation of the reaction mixtures at 10,000 rpm for 10 min, the supernatant was collected, and absorbance at 310 nm was measured and plotted.

#### Effect of the reaction time

Cell-free supernatant (10 mL) was mixed with 10 mL of 100 mM ZnSO₄ solution and incubated at 37 °C under light and stirring conditions (120 rpm) for varying time intervals (24, 48, 72, and 96 h). After centrifugation at 10,000 rpm, the absorbance of the collected samples was measured at 310 nm to determine the optimal reaction time for nanoparticle synthesis [36].

#### Effect of ZnSO_4_ concentration

Various ZnSO₄ concentrations (50–300 mM) were tested to evaluate their impact on ZnNP production [37]. A volume of 10 ml of ZnSO₄ was mixed with 10 ml of cell free supernatant at pH 7.0. The mixtures were incubated at 37 °C under light conditions with continuous stirring at 120 rpm. After 72 h, absorbance was measured at 310 nm (λ₃₁₀) to evaluate zinc nanoparticle (ZnNP) formation.

#### Effect of cell culture supernatant

To assess the effect of cell-free supernatant volume on ZnNP synthesis, varying volumes of supernatant (5, 10, 15, 20, and 25 ml) were mixed with 10 ml of ZnSO₄ solution (pH 7.0). The total reaction volume was adjusted to 35 ml using sterile LB medium. The mixtures were incubated at 37 °C under light conditions with continuous stirring at 120 rpm for 72 h. Following incubation, samples were collected, and absorbance at 310 nm (λ₃₁₀) was measured to evaluate nanoparticle formation. Results were analyzed by plotting the maximum absorbance recorded for each treatment.

#### Effect of pH

The pH of the reaction mixture was adjusted to 4, 6, 7, 8, and 10. Reactions were conducted under previously established conditions specific to each microorganism and incubated for 72 h, after which absorbance was measured at 310 nm [38]. Further pH optimization was performed using citrate buffer (pH 3.3, 3.7, 4.0, 4.3, and 4.7) and Tris-HCl buffer (pH 7.3, 7.6, 8.0, 8.3, and 8.6). The reactions were then repeated under identical conditions.

#### Effect of medium

The effect of low-salt LB broth on zinc nanoparticle (ZnNP) production was evaluated in comparison to standard LB broth. Standard LB medium contained 1.0% tryptone, 0.5% yeast extract, and 1.0% sodium chloride (NaCl), whereas the low-salt LB medium had the same composition except with a reduced NaCl concentration of 0.1%. Cultures were incubated under identical conditions, and ZnNP synthesis was assessed by measuring absorbance after 72 h at λ₃₁₀.

#### Effect of temperature

Reactions were conducted at 30, 37, 40, and 45 °C under optimized conditions specific to each microorganism. After 72 h of incubation, absorbance at 310 nm was measured to assess the temperature dependence of nanoparticle production [39].

### Lyophilization

The culture supernatant obtained under optimized conditions was lyophilized using a Benchtop Pro with Omnitronics™ freeze dryer (SP Scientific, Virtis, USA**)**. The lyophilization process was carried out at a chamber pressure of 0.12 mbar and a condenser temperature of − 55 °C for 24 h. This process yielded powdered zinc nanoparticles (ZnNPs), which were subsequently used for further physicochemical characterization and application studies [40].

### Characterization of biosynthesized ZnNPS

#### TEM analysis

Nanoparticles synthesized using bacterial supernatants were characterized by TEM to determine their size, and morphology [41]. A small drop (~ 10 µl) of the nanoparticle suspension was carefully placed onto a gold-coated carbon-coated copper grid and allowed to air-dry at room temperature for 10–15 m. To preserve the native structure and avoid introducing artifacts, no staining or chemical treatment was applied prior to imaging. The dried samples were then examined using a JEOL JEM-1400 Plus TEM (JEOL Ltd., Japan) operating at an accelerating voltage of 120 kV. Images were captured at various magnifications to assess particle size, shape, and aggregation state. Measurements were performed using image analysis software to quantify particle dimensions.

#### X-ray diffraction (XRD) analysis

The ZnNPs sample was dried for XRD analysis, conducted in transmission mode using Bruker D2 PHASER instrument. XRD was performed in the 2θ range of 20–80˚ with Cu Kα radiation (λ = 1.5406 Å) at 30 kV and 15 mA. Diffraction patterns were recorded as step-scans, with parameters including 2θ angle, step size (0.005°), and count time (0.05–1 s) set for analysis.

The crystallite size (D) was calculated using the Scherrer equation:

D = (K × λ) / (β × cos θ),

where D is the size in nanometers, K is the shape factor (0.9), λ is the X-ray wavelength (0.15406 nm for Cu Kα), β is the peak width at half maximum in radians, and θ is the Bragg angle in radians (half of the 2θ value).

#### Energy dispersive X-ray spectroscopy (EDX) analysis

EDX is used to identify the elemental composition of a sample. It works by analyzing X-rays emitted from the sample when bombarded with charged particles. Each element has a unique atomic structure, allowing for distinct identification of its characteristic X-rays. The analysis was performed on lyophilized ZnNP powder using Oxford instruments attached to SEM.

### Fourier transform infrared (FTIR) spectroscopy

The interaction between biomolecules and ZnNPs was analyzed by Fourier Transform Infra-Red spectroscopy. The synthesized ZnNPs sample was lyophilized and all spectra were obtained using pressed pellets of the prepared samples in potassium bromide (KBr). The FTIR spectrum was analysed by Bruker Tensor37 instrument. The measurements were carried out in the range of (350–4400) cm^− 1^ [42]. Baseline correction and peak deconvolution were conducted using OPUS software to ensure accurate identification of vibrational modes.

### Biotechnological applications of bacterial ZnNPs

#### Antimicrobial dose-response of ZnNPs

ZnNPs were tested against Gram-positive, Gram-negative bacteria, and yeast using the agar well diffusion method. The zones of inhibition were measured to assess antimicrobial effectiveness, based on the average of three biological replicates [43].

*Escherichia coli ATCC 25,922 (E. coli*), *Klebsiella pneumoniae* ATCC 13,883 *(K. pneumoniae)*,* Staphylococcus aureus* ATCC 25,923 *(S. aureus)*,* Staphylococcus epidermidis* ATCC 12,228 *(S. epidermidis*), *Enterococcus faecalis ATCC 29,212 (E. faecalis)*, and *Candida albicans* ATCC 10,231 (*C. albicans*) were used as test strains.

The strains were grown in LB broth at 37 °C for 24 h, then centrifuged (5000 rpm, 5 min), washed with PBS (pH 7.4), and re-suspended in fresh LB medium. Sterile agar plates were swabbed with 100 µl of the suspension and incubated at 37 °C for 60 min to allow adherence before 5-mm wells were created. Zinc-based nanoparticles (ZnNPs), prepared at 25, 50, and 100 µg/ml in sterile distilled water and dispersed by mild ultrasonication (5–10 min), were added to the wells. Vancomycin, Gentamicin, and Fluconazole at a concentration of 100 µg/ml each were used as positive controls against Gram-positive bacteria, Gram-negative bacteria, and *Candida* species. Sterile distilled water was used as a negative control. Plates were incubated for 24 h at 37 °C, after which the diameters of inhibition zones were measured in millimetres to evaluate antimicrobial activity and dose-response effects.

### Assessing ZnNPs effects on sludge microbiota via optical density (OD) measurement

ZnNPs were tested for their ability to treat sludge from wastewater treatment plants by measuring growth inhibition of microorganisms in the sludge. In this experiment, one gram of sludge sample was added to 50 ml of distilled water and stirred for 3 h. Then, 1 ml of the resulting suspension was added to flasks containing 50 ml of LB broth medium. Different concentrations of ZnNPs (12.5, 25, and 50 µg/ml) were individually tested, with one flask kept as an untreated control (without ZnNPs). The selected concentrations were maintained below the established safety threshold of ≤ 100 µg/mL [44]. The samples were incubated under standardized conditions (37 °C, continuous light exposure, 120 rpm) for 48 h. Microbial growth was evaluated by measuring the optical density at 600 nm (OD₆₀₀) after 24 and 48 h. The results were compared to the untreated control [45], and growth inhibition was expressed as percentage reduction relative to the control.

### Analysis of experimental data

Statistical analysis was conducted using analysis of variance (ANOVA) with CoStat software. Differences between means were evaluated using the Least Significant Difference (LSD) test at a significance level of *P* ≤ 0.05. All values represent the average of three independent biological replicates.

## Results

### Isolation and screening of ZnNP-producing microorganisms

Preliminary screening was carried out to evaluate the potential of isolated microorganisms for the extracellular synthesis of zinc-based nanoparticles (ZnNPs). A total of 40 microbial isolates were tested by adding 100 mM ZnSO₄ to 72-hour-old culture supernatants, followed by incubation at 37 °C and 120 rpm for 72 h. The formation of ZnNPs was initially indicated by a visible color change from colorless to milky white or pale yellow under ambient light conditions, in the presence of ZnSO₄. No color change occurred in control samples containing sterile medium without microbes and ZnSO₄ solution.

**Fig. 2:**
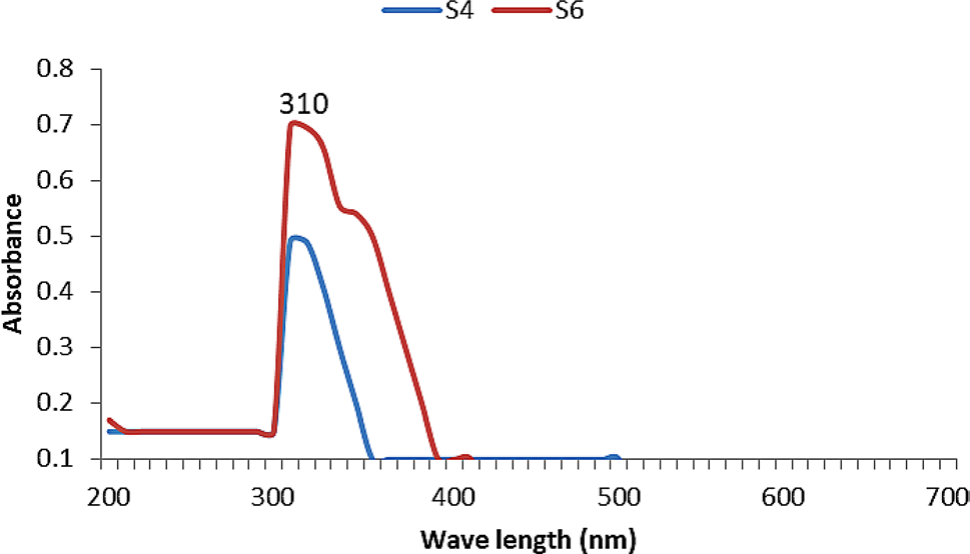
UV–Visible scan spectra of ZnNPs synthesized by strains S4 and S6, showing a maximum absorption peak at 310 nm

**Fig. 3:**
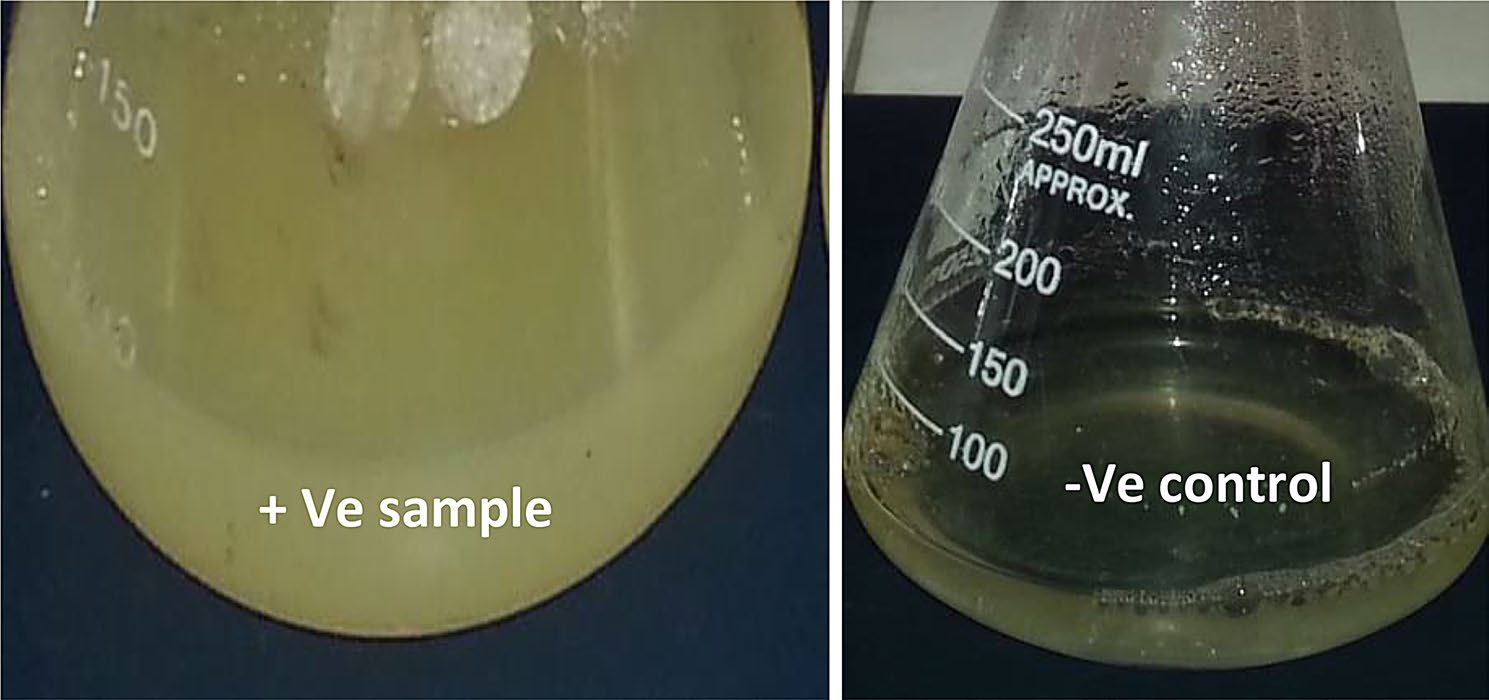
Visible white-creamy color change in the positive sample containing cell-free supernatant reacted with 100 mM ZnSO₄. No color change was observed in the negative control (non-inoculated medium with 100 mM ZnSO₄)

This color change is attributed to the formation of either zinc oxide (ZnO) through oxidation or zinc sulfide (ZnS) via the reaction of zinc ions with sulfide ions, or a combination of both, resulting in ZnO–ZnS composite nanoparticles. UV–Visible spectrophotometric analysis (200–800 nm) revealed that only 8 out of 40 isolates exhibited significant absorbance peaks, as summarized in Table 1. Among these, isolates S4 and S6 showed the highest absorbance at approximately 310 nm **(**Fig. 2**)**, corresponding to the characteristic surface plasmon resonance of zinc-based nanoparticles, particularly ZnO and ZnS. The associated visual color changes for these isolates are shown in Fig. 3, further confirming the successful formation of zinc nanoparticles (ZnNPs).

**Table 1:** UV–Visible spectral analysis of Zn nanoparticle production by bacterial isolates, showing collection source, λmax, and absorbance intensity for each isolate

Isolate code	Collection place	Source for isolation	Maximum absorbance peaks (λmax)	Absorbance intensity
**S4**	**Alexandria**	Mango - rhizoshere	**310**	**0.4910**
S9	El-Behira	Peach rhizosphere	418	0.2639
S8—R	Alexandria	Peach rhizosphere	418	0.2599
S4cr	Alexandria	Mango rhizosphere	419	0.4525
S8—B	Alexandria	Peach rhizosphere	418	0.4049
S2	Alexandria	Apple rhizosphere	280	00.142
**S6**	**Alexandria**	Peach -rhizosphere	**310**	**0.6973**
S5	Alexandria	Apple rhizosphere	260	00.019

### Identification of the selected isolates

**Fig. 4:**
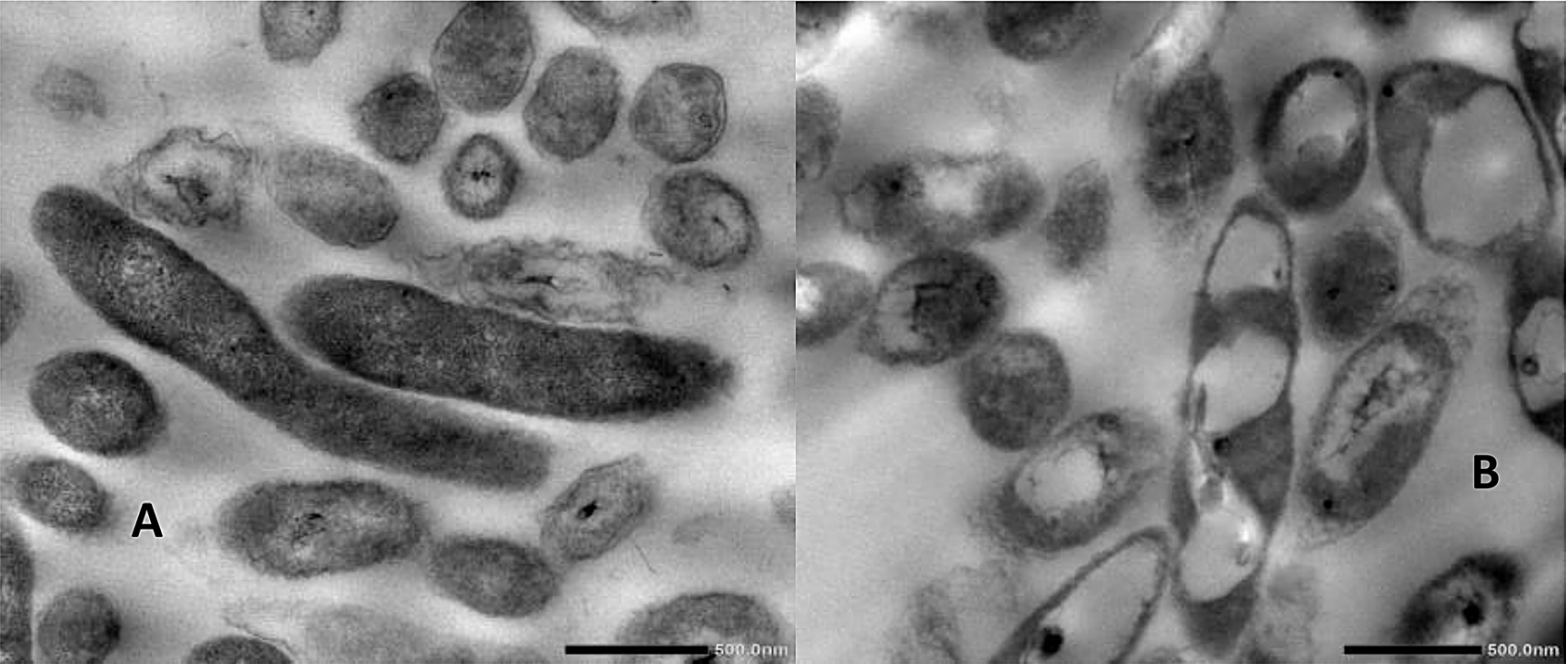
TEM micrographs at 500 nm scale for the two selected isolates: S4 **(A)** and S6 **(B)**

Initial bacterial identification was performed based on phenotypic characteristics, including colony morphology, pigmentation, and biochemical profiling. TEM analysis revealed that strain S4 exhibited curved rod-shaped cells with extracellular material, while strain S6 showed the presence of internal vacuoles or inclusion bodies **(**Fig. 4A, B**)**.

Further identification using MALDI-TOF mass spectrometry confirmed the isolates as *Achromobacter* sp. (S4) and *Pseudomonas* sp. (S6), based on ribosomal RNA protein markers.

### Sequencing of *16 S RNA* gene

The purified *16 S rRNA* PCR products for strains S4 and S6 were sequenced and analyzed using BLAST. The sequences showed 97.84% similarity to *Achromobacter spanius* strain E2BF6-2 (Accession No. KM604983.1) and 98.05% similarity to *Pseudomonas resinovorans* strain MOB-449 (Accession No. MK011872.1). These sequences were submitted to GenBank under accession numbers PP593876 (S4) and PP535560 (S6). Morphologically, S4 retained the characteristic curved rod shape of *Achromobacter spanius*, while S6 exhibited inclusion bodies, presumed to be polyhydroxyalkanoates (PHA).

### Factors affecting ZnNP formation

**Fig. 5:**
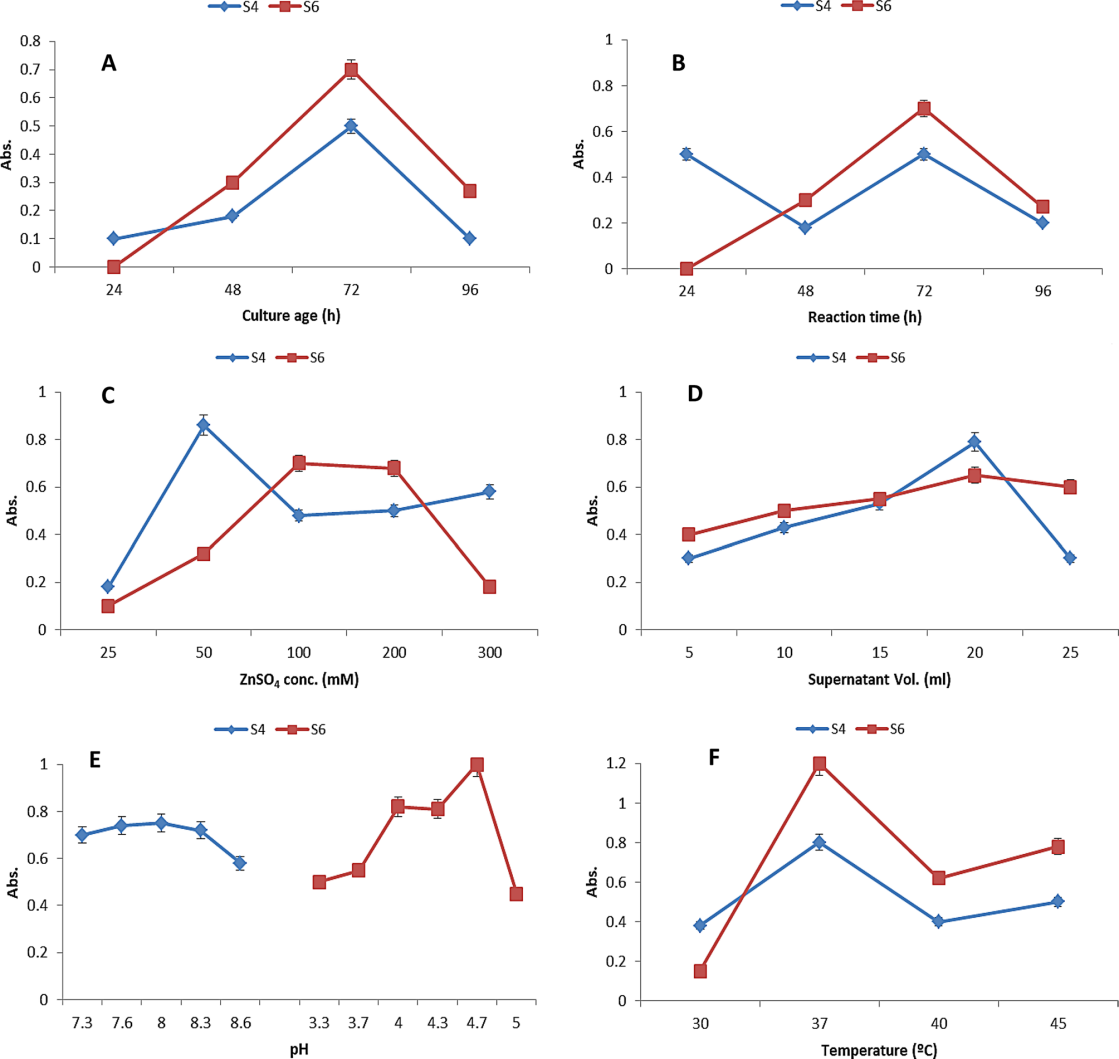
Monitoring the maxium absorbance reading of reaction product at λ310 under different conditions for the selected strains (S4&S6). Culture age **(A)**, reaction time **(B)**, ZnSO_4_ concentrations **(C)**, supernatant of a cell **(D)**, pH **(E)** and temperature **(F)**

Optimization of various physicochemical parameters was carried out to enhance zinc nanoparticle (ZnNP) synthesis by *Achromobacter spanius* (S4) and *Pseudomonas resinovorans* (S6), using UV–Visible spectroscopy at 310 nm as the primary indicator of nanoparticle formation. Both isolates showed maximum absorbance after 72 h of incubation, with values of 0.5 for S4 and 0.7 for S6, indicating this as the optimal culture age (Fig. 5A). Reaction time also influenced nanoparticle yield, with peak absorbance again observed at 72 h—0.4821 for S4 and 0.6823 for S6 (Fig. 5B). Zinc sulfate concentration significantly affected nanoparticle synthesis, with optimal results at 50 mM for S4 (absorbance: 0.8322) and 100 mM for S6 (absorbance: 0.6937) (Fig. 5C). Varying the volume of culture supernatant revealed a trend of increasing ZnNP intensity with increasing volume, followed by a decline; the best results were observed using 100 mM ZnSO₄ for S6 and 50 mM for S4 (Fig. 5D).

pH was found to be a crucial factor influencing nanoparticle synthesis. Initial screening over a pH range of 4 to 10 showed maximum nanoparticle intensity at pH 4 for S6 and pH 8 for S4. Further refinement using citrate buffer (pH 3.3–5.0) for S6 and Tris-HCl buffer (pH 7.3–8.6) for S4 confirmed the optimal pH values to be 4.7 and 8.0, respectively (Fig. 5E). Additionally, salt concentration in the culture medium played a role, as full-strength LB medium yielded higher absorbance values—0.8153 for S4 and 1.0714 for S6—compared to LB with half the salt content (data not shown). Temperature also influenced ZnNP production, with both isolates showing maximum synthesis at 37 °C (Fig. 5F). A summary of all optimal parameters is presented in Table 2. These conditions were used for nanoparticle extraction, involving centrifugation at 10,000 rpm and subsequent lyophilization of the supernatant.

**Table 2:** Summary of optimized conditions for Zn nanoparticle synthesis by *A. spanius* strain S4 and *P. resinovorans* strain S6, including culture parameters, reaction settings, and final absorbance at λmax

Conditions	A. spanius strain S4	*P*. resinovorans strain S6
Culture age	72 h	72 h
ZnSO_4_ concentration	0.5 M	0.1 M
Supernatant volume	20 ml	20 ml
pH	8 (basic)	4.7 (acidic)
Temperature	37 ºC	37 ºC
Medium	L.B	L.B
Incubation period for the reaction	72 h	72 h
Final absorbance at λmax	**0.8153**	**1.0714**
Fold increase	**1.66**	**1.53**

### Characterization of Zn nanoparticles

Zinc nanoparticles (ZnNPs) synthesized by *Achromobacter spanius* (S4) and *Pseudomonas resinovorans* (S6) were characterized using a combination of techniques including Transmission Electron Microscopy (TEM), Energy-Dispersive X-ray Spectroscopy (EDX), X-ray Diffraction (XRD), and Fourier-Transform Infrared Spectroscopy (FTIR).

**Fig. 6:**
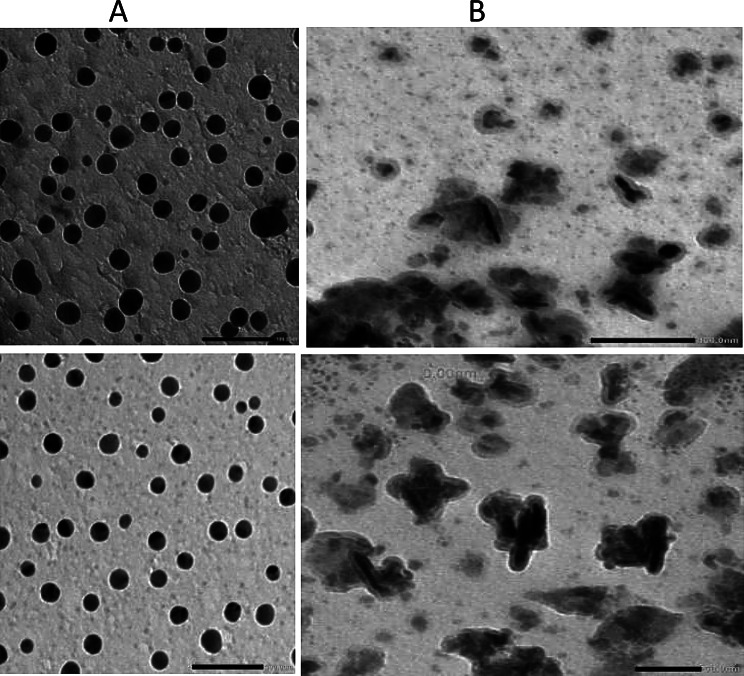
Transmission electron microscopy (TEM) images of biosynthesized ZnSNPs and ZnO–ZnSNPs displaying distinct morphologies: spherical nanoparticles produced by *A. spanius* strain S4 **(A)**, and rod-shaped nanoparticles produced by *P. resinovorans* strain S6 **(B)**. Scale bar = 100 nm. The particle sizes ranged from 14.1 to 29 nm for S4 and 3.93 to 43.9 nm for S6

TEM analysis revealed that the ZnNPs produced by S4 were spherical in shape with particle sizes ranging from 14.1 to 29 nm **(**Fig. 6A**)**, while S6-derived nanoparticles appeared rod-shaped, with sizes varying between 3.93 and 43.9 nm **(**Fig. 6B**)**.

**Fig. 7:**
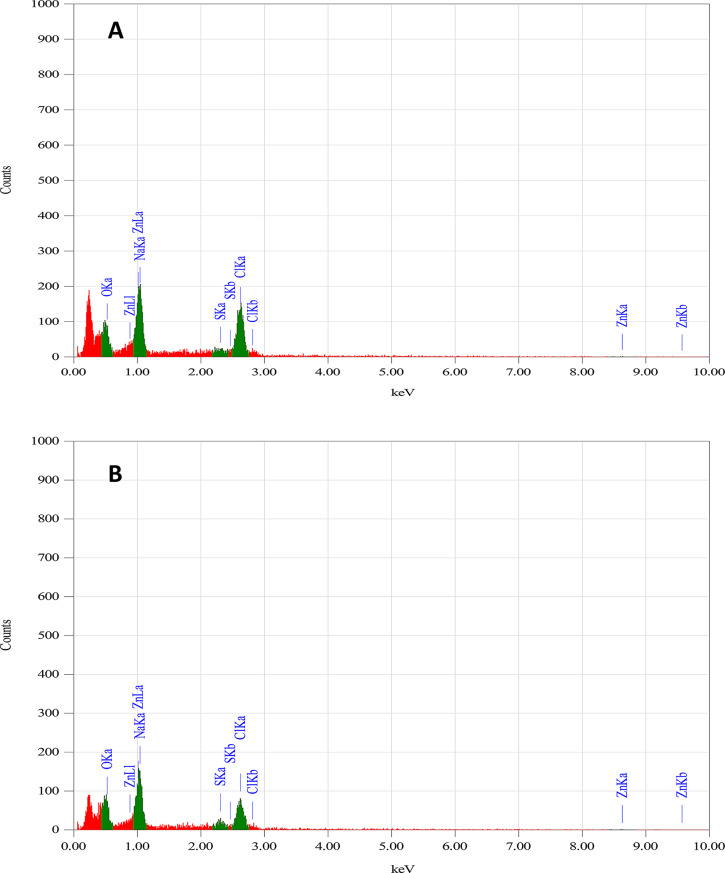
EDX spectra of ZnS nanoparticles synthesized by *A. spanius* strain S4 **(A)** and ZnO–ZnS nanoparticles synthesized by *P. resinovorans* strain S6 **(B)**

EDX analysis confirmed the elemental composition of the biosynthesized nanoparticles. Strain S4 predominantly produced ZnS nanoparticles, whereas strain S6 synthesized a hybrid of ZnS and ZnO **(**Fig. 7A, B**)**. Distinct zinc (Zn) peaks appeared at approximately 0.9–1.0 keV (Zn Lα) and 8.7–9.7 keV (Zn Kα), confirming zinc as the primary element in both nanoparticle types. Additional signals for oxygen (O), sodium (Na), sulfur (S), and chlorine (Cl) were also detected, likely derived from culture media or biomolecular capping agents.

**Fig. 8:**
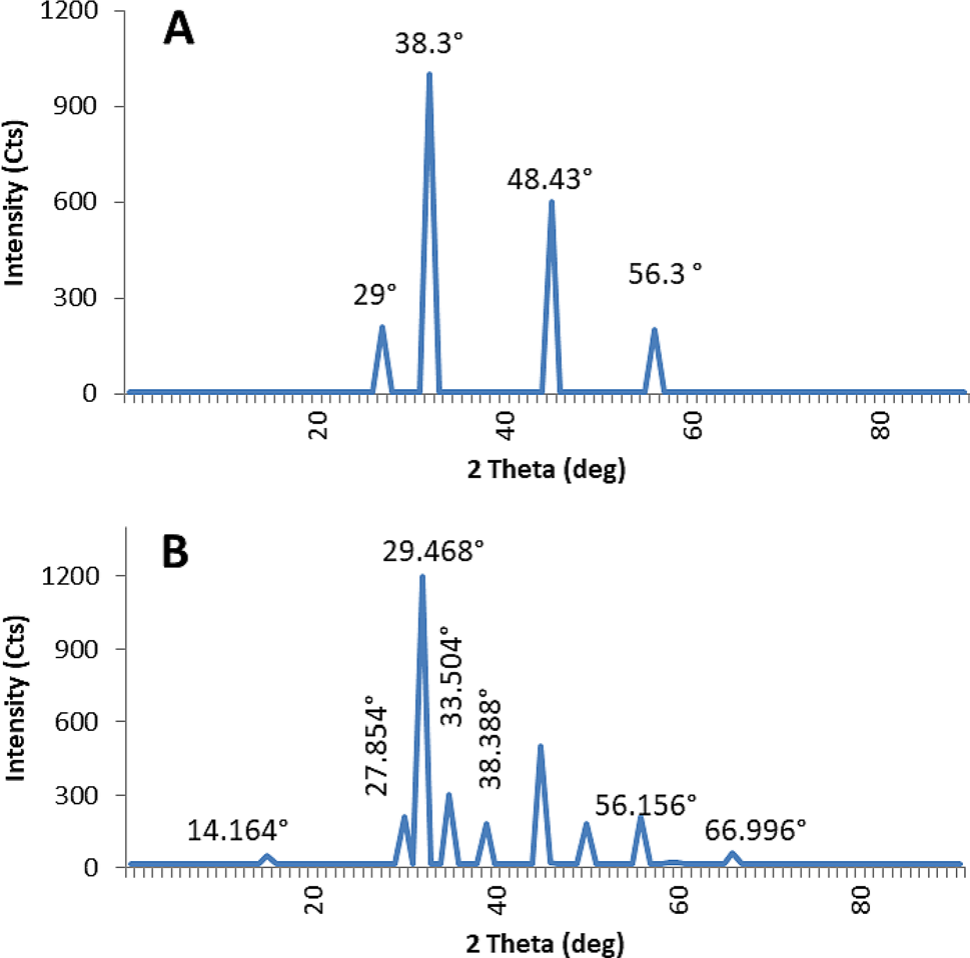
XRD analysis of ZnS nanoparticles synthesized by *A. spanius* strain S4 **(A)** and ZnO–ZnS nanoparticles synthesized by *P. resinovorans* strain S6 **(B)**

**X**-ray diffraction (XRD) analysis revealed that the ZnNPs synthesized by isolate S4 exhibited distinct diffraction peaks at 29.00°, 38.30°, 48.43°, and 56.30°, corresponding to the (111), (200), (220), and (311) planes of a cubic zinc sulfide (ZnS) structure, as referenced by JCPDS card no. 05-0566 **(**Fig. 8A**)**. The average crystallite size was estimated to be 27.7 nm using the Scherrer equation.

For isolate S6, the X-ray diffraction (XRD) pattern exhibited distinct diffraction peaks at 4.71°, 27.85°, 29.47 °, 14.164 ° and 38.388 ° which were indexed to the (2), (103), (105), (6), and (16) crystallographic planes. These reflections correspond to the hexagonal wurtzite structure of zinc oxide (ZnO), in accordance with JCPDS card no. 36-1451. In addition, diffraction peaks observed at 33.50°, 56.16°, and 66.99°, indexed to the (111), (220), and (311) planes, respectively, were characteristic of the cubic zinc sulfide (ZnS) phase **(**Fig. 8B**)**.

The simultaneous presence of ZnO and ZnS phases was further supported by overlapping peaks at 27.85° and 29.47°, indicating the formation of a ZnO–ZnS nanocomposite. The average crystallite sizes, calculated using the Scherrer equation, were found to be 23.5 nm for the ZnO phase and 20.7 nm for the ZnS phase.

**Fig. 9:**
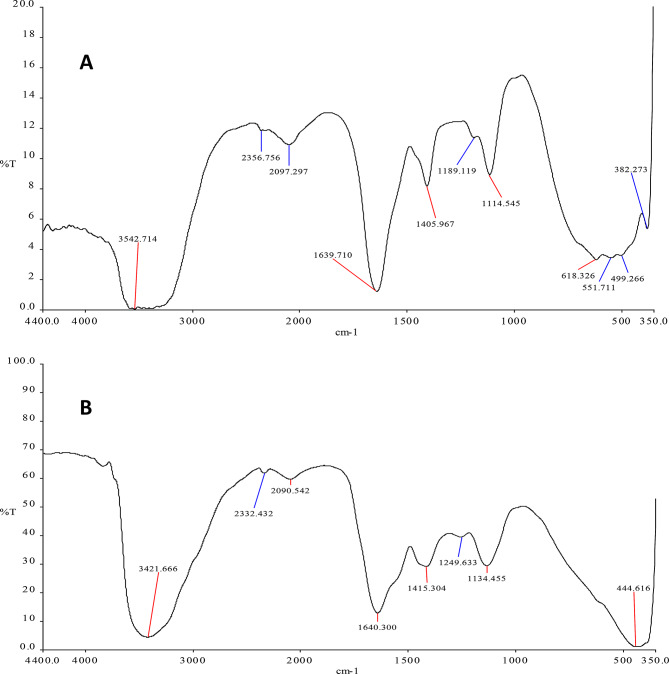
FTIR spectra of ZnS nanoparticles synthesized by *A. spanius* strain S4 **(A)** and ZnO–ZnS nanoparticles synthesized by *P. resinovorans* strain S6 **(B)**

FTIR spectroscopy provided insights into the functional groups involved in the reduction and stabilization of zinc-based nanoparticles. For nanoparticles synthesized by S4 **(**Fig. 9A**)**, a broad absorption band at 3542.71 cm⁻¹ was attributed to O–H stretching vibrations, indicating the presence of hydroxyl groups from water, alcohols, or phenolic compounds. Peaks at 2356.76 cm⁻¹ and 2097.30 cm⁻¹ were assigned to C ≡ O and C–O stretching vibrations, respectively, likely arising from carbonyl-containing organic compounds or microbial metabolites.

A prominent band at 1639.30 cm⁻¹ corresponded to the C = O stretching vibration of amide II, suggesting the involvement of proteins in nanoparticle stabilization, potentially through electrostatic or covalent interactions. The band observed at 1405 cm⁻¹ was indicative of O–H bending, while the peak at 1114.55 cm⁻¹ corresponded to C–C–O stretching, typically found in alcohols, ethers, or polysaccharides. Additionally, a low-frequency band at 382.27 cm⁻¹ and absorption in the region of 449.2–618.2 cm⁻¹ confirmed the formation of ZnS nanoparticles, corresponding to Zn–S stretching modes.

For nanoparticles synthesized by isolate S6 **(**Fig. 9B**)**, the FTIR spectrum showed a broad O–H stretching band at 3421.67 cm⁻¹, indicative of hydroxyl-containing compounds such as alcohols or bound water. A distinct absorption at 2332.43 cm⁻¹ was assigned to C–H stretching vibrations from aromatic aldehydes, suggesting the presence of aromatic organic molecules. The peak at 1640.30 cm⁻¹, similar to that observed in S4, was attributed to C = O stretching in amide II, again implying protein involvement as potential capping or reducing agents.

A peak at 1415 cm⁻¹ corresponded to the asymmetric stretching of carboxylate (COO⁻) groups, possibly derived from residual acetate or organic acids. The band at 1134 cm⁻¹ was associated with C–O stretching vibrations of alcohols or polysaccharides. A strong absorption band at 444.62 cm⁻¹ was characteristic of Zn–O and Zn–S stretching, confirming the formation of ZnO–ZnS composite nanoparticles.

These spectral features collectively suggest that biomolecules such as proteins, carboxylic acids, and phenolic compounds played crucial roles in the biosynthesis and stabilization of ZnNPs in both samples.

A summary of the FTIR spectral data for both isolates is provided in Table 3.

**Table 3:** FTIR band assignments of ZnS NPs and ZnS-ZnO NPs synthesized by isolates S4 and S6

Wavenumber (cm⁻¹)	Sample	Assignment	Functional Group / Interpretation
3542.71	S4	O–H stretching	Hydroxyl groups (alcohols, phenols, water)
2356.76	S4	C ≡ O or C–O stretching	Carbonyl-containing compounds, possible CO₂ adsorption
2097.30	S4	C–O stretching	Aldehyde or ether groups; microbial metabolites
1639.30	S4	C = O stretching (amide II)	Proteins/peptides involved in nanoparticle stabilization
1405.00	S4	O–H deformation	Phenolic O–H bending or carboxylic acids
1114.55	S4	C–C–O stretching	Alcohols, ethers, or polysaccharides
382.27	S4	Stacked base vibration	Possibly related to aromatic rings or nitrogenous bases
449.2–618.2	S4	Zn–S stretching	Formation of ZnS nanoparticles
3421.67	S6	O–H stretching	Hydroxyl groups (from alcohols, phenols, or water)
2332.43	S6	C–H stretching (aromatic aldehydes)	Aromatic aldehydes or volatile organic compounds
1640.30	S6	C = O stretching (amide II)	Protein-based capping or reducing agents
1415.00	S6	COO⁻ asymmetric stretching	Carboxylate groups, likely from acetate or microbial acids
1134.00	S6	C–O stretching	Alcohols, ethers, or metal–oxygen bonding
444.62	S6	Zn–O and Zn–S stretching	Confirmation of ZnO–ZnS nanoparticle formation

### Antimicrobial and sludge treatment efficacy of biosynthesized Zn based NPs

**Fig. 10:**
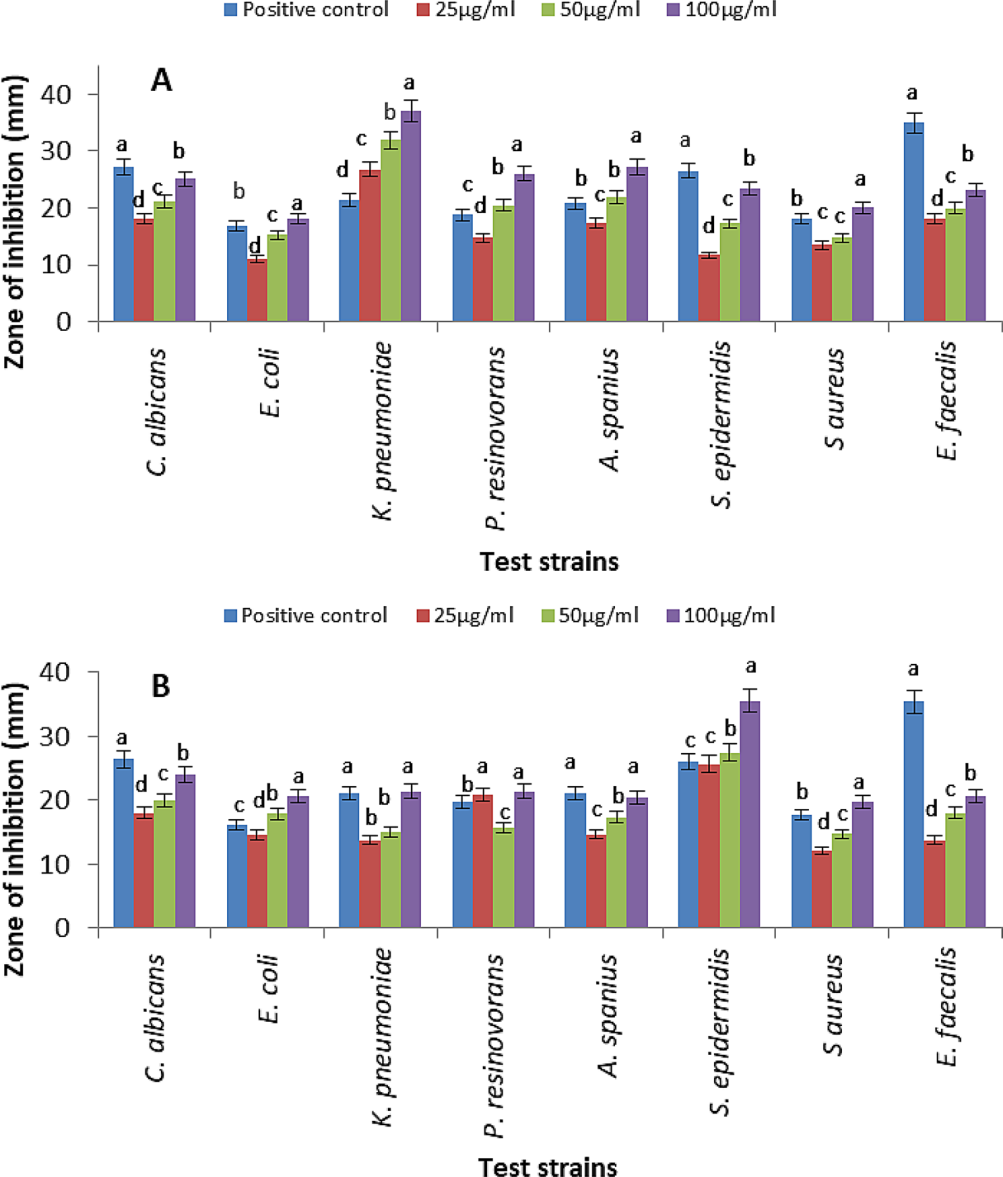
Antimicrobial activity of ZnSNPs from *A. spanius* S4 (**A**) and ZnS–ZnO NPs from *P. resinovorans* S6 (**B**) at 25, 50, and 100 µg/ml. Vancomycin, Gentamicin, and Fluconazole (100 µg/ml) served as positive controls against Gram-positive bacteria, Gram-negative bacteria, and *Candida* spp., respectively. Data represent mean ± SD of inhibition zones from three biological replicates. Different lowercase letters indicate significant differences (*P* ≤ 0.05) among treatments relative to the positive control

The antimicrobial activity of biosynthesized ZnNPs from *Achromobacter spanius* (S4) and *Pseudomonas resinovorans* (S6) was evaluated against a panel of pathogenic bacteria and fungi. Antibacterial testing revealed that ZnS nanoparticles derived from S4 exhibited the highest inhibitory effect against *Klebsiella pneumoniae* at a concentration of 100 µg/ml **(**Fig. 10A**)**. In contrast, the ZnO-ZnS nanoparticles synthesized by S6 demonstrated the strongest activity against *S. epidermidis* at the same concentration **(**Fig. 10B**)**.

In both cases, the nanoparticles showed greater antimicrobial efficacy compared to the positive control antibiotic, with the exception of *Enterococcus faecalis*, which remained less susceptible.

**Fig. 11:**
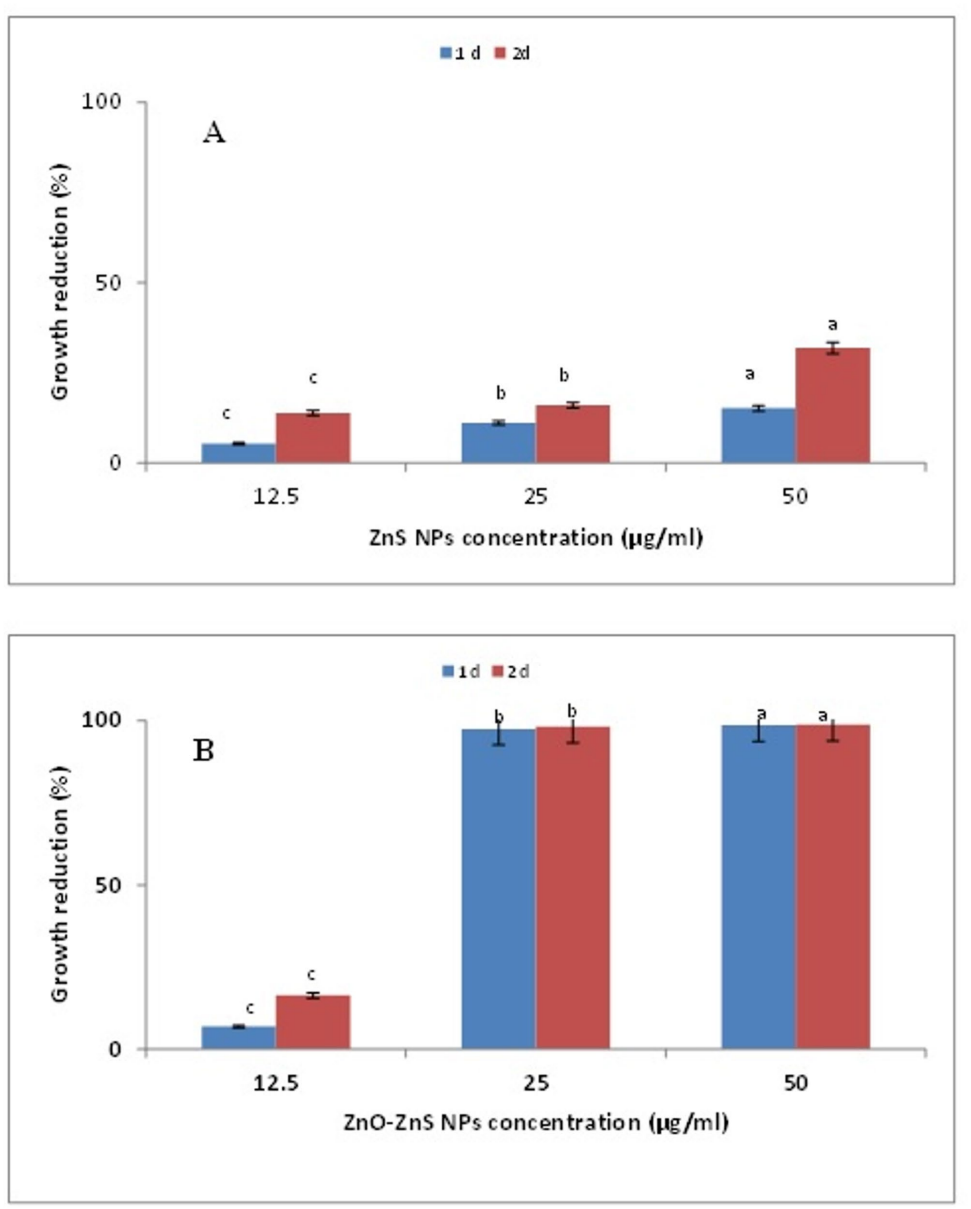
Percentage growth reduction of sludge microorganisms treated with ZnS nanoparticles (NPs) from *A. spanius* S4 (**A**) and ZnO–ZnS NPs from *P. resinovorans* S6 (**B**) at concentrations of 12.5, 25, and 50 µg/ml. Growth reduction was assessed based on optical density measurements over two days and compared to the untreated control. Different lowercase letters indicate statistically significant differences among treatments (*P* ≤ 0.05)

In addition to antimicrobial screening, the sludge treatment potential of the nanoparticles was evaluated. ZnS nanoparticles (ZnS NPs) from S4 and mixed-phase ZnS–ZnO nanoparticles (ZnS–ZnO NPs) from S6 were applied to sludge samples at various concentrations, and microbial growth was monitored over two days. Notably, after one day, the ZnS–ZnO NPs from S6 caused a marked reduction in microbial growth at 25 µg/ml, outperforming the single-phase ZnS NPs from S4 **(**Fig. 11A, B**)**, thus demonstrating higher treatment efficiency.

## Discussion

The extracellular synthesis approach employed in this study presented a significant advantage over intracellular methods, primarily due to the ease of nanoparticle recovery directly from the culture supernatant, without requiring cell disruption or complex purification steps, making the process more efficient and scalable. The strong UV-Visible absorbance peak observed at approximately 310 nm in isolates S4 and S6 aligns with previous studies identifying this wavelength as a characteristic feature of ZnO nanoparticles [38, 46]. ZnS nanoparticles, on the other hand, typically exhibit absorbance in the 280–320 nm range—most commonly between 290 and 310 nm—due to their wide band gap (~ 3.7 eV) and associated electronic transitions. The exact peak positions can vary depending on particle size and crystallinity, as noted by Segura et al. [47]. The broad nature of the absorbance peaks reported in the literature are often attributed to a wide particle size distribution, where quantum size effects result in varying optical properties and shifts in peak position [47, 48]. A visual color change to milky white or pale yellow was noted during biosynthesis ZnNPs using ZnSO₄ and bacterial culture supernatants. This transformation serves as a rapid, cost-effective indicator of nanoparticle formation and is attributed to the interaction of Zn²⁺ ions with microbial metabolites, leading to the formation of colloidal ZnS or ZnO nanoparticles [48–50]. In certain cases, the biological reduction of Zn²⁺ may also lead to the generation of metallic Zn⁰ nanoparticles [51]. The biosynthesis of hybrid ZnO-ZnS nanoparticles (ZnO-ZnS NPs) is hypothesized to occur via a combination of bioreduction, complexation, and nucleation processes. Enzymes such as nitrate and sulfite reductases likely facilitate the reduction of Zn²⁺ and sulfur species. ZnO forms via interaction with hydroxyl groups, while ZnS forms through reactions with biologically produced hydrogen sulfide (H₂S). Under specific pH and redox conditions, these processes promote co-nucleation, resulting in hybrid ZnO-ZnS structures. Bacterial metabolites—including proteins and polysaccharides—act as capping agents, stabilizing the nanocomposites and contributing to their enhanced bioactivity [52, 53].The absence of color change in negative controls affirms that nanoparticle synthesis is biologically mediated rather than chemically spontaneous. This finding aligns with observations of Elamawi et al. [54], Smitha et al. [55], and Ahluwalia et al. [56], who reported that colorimetric shifts during nanoparticle formation only occur in the presence of biological agents. Hanisha et al. [57] highlighted the crucial role of biological activity in driving the color change during algae-mediated silver nanoparticle synthesis, underscoring the fundamental importance of biological processes in nanoparticle biosynthesis.

While phenotypic identification remaining a foundational, its accuracy can be limited by environmental and physiological factors [58]. To enhance taxonomic resolution, this study incorporated genotypic methods alongside phenotypic observations. MALDI-TOF mass spectrometry, which identifies bacteria based on ribosomal protein fingerprints, provided rapid and reliable strain identification [28, 29]. These findings were corroborated by transmission electron microscopy (TEM), which revealed morphological traits characteristic of the *Achromobacter* and *Pseudomonas* genera. Further validation was obtained through *16 S rRNA* gene sequencing, a gold-standard technique for bacterial classification [59–61]. High sequence similarity confirmed the identity of isolate S4 as *Achromobacter spanius* and isolate S6 as *Pseudomonas resinovorans*. Additionally, the observation of polyhydroxyalkanoate (PHA)-like inclusion bodies in S6 is consistent with known metabolic features of *Pseudomonas* species, highlighting their broader biotechnological potential [62, 63]. The selection of *Achromobacter* and *Pseudomonas* spp. was grounded in their proven capabilities in metal ion reduction and nanoparticle biosynthesis. Both genera harbor diverse redox-active enzymes—such as nitrate reductases, NADH-dependent reductases, and oxidoreductases—which are instrumental in converting metal salts into nanoparticles. *Pseudomonas* spp. are particularly efficient in producing extracellular enzymes and secondary metabolites that promote both the synthesis and stabilization of metal nanoparticles including ZnO, Ag, and Au [64, 65]. Although *Achromobacter* spp. are less frequently reported for NP biosynthesis, emerging studies highlight their resilience in metal-contaminated environments and their capacity for redox transformations via membrane-bound reductases [66], making them promising yet underexplored candidates for green nanotechnology.

Moreover, both isolates demonstrated biofilm formation and secretion of extracellular polymeric substances (EPS), which facilitate nanoparticle capping and prevent agglomeration—key factors for maintaining nanoscale size and colloidal stability [65].

Controlling the synthesis of zinc nanoparticles (ZnNPs) with precision remains a challenge, as key parameters such as temperature, pH, metal ion concentration, and incubation time significantly influence nanoparticle characteristics [67]. In the present study, these variables were systematically optimized for *Achromobacter spanius* (S4) and *Pseudomonas resinovorans* (S6) to enhance ZnNP production.

The formation of ZnNPs was confirmed by UV–Visible spectroscopy, with both isolates exhibiting strong absorbance at approximately 310 nm—a finding consistent with previous reports [47, 48]. Nanoparticle synthesis was observed after 72 h of incubation, aligning with the observations of Ashajyothi et al. [34], who also identified 72 h and 100 mM ZnSO₄ as optimal conditions for microbial ZnNP biosynthesis.

Among the tested variables, pH emerged as a critical factor influencing nanoparticle yield and morphology. The results corroborate previous studies indicating that Zn solubility increases under acidic conditions, while higher pH values can lead to partial nanoparticle disaggregation [68, 69]. In this study, the optimal pH was 4.7 for *P. resinovorans* (S6) and 8.0 for *A. spanius* (S4). These findings are in agreement with Nagarajan & Kuppusamy [37], who observed complete reduction of zinc nitrate to ZnNPs at pH 8 using seaweed extract, and Mohammadi & Ghasemi [70], who found basic pH essential for ZnO nanoparticle synthesis using cherry extract. Płaza et al. [71] similarly reported that silver nanoparticles synthesized under acidic conditions tend to be smaller in size. Additionally, Sadollahkhani et al. [72] demonstrated that the adsorption efficiency of Rose Bengal on ZnO-based nanoparticles peaked at pH 4, highlighting how surface interactions are also pH-dependent.

Salt concentration in the growth medium significantly influences the biosynthesis of zinc-based nanoparticles (ZnNPs). In this study, full-strength LB medium yielded higher ZnNP production compared to reduced-salt formulations, likely due to enhanced bacterial growth, increased metabolic activity, and upregulation of redox enzymes responsible for nanoparticle synthesis. Sodium chloride (NaCl), a key component of LB medium, contributes to enzyme stability and facilitates the effective reduction of zinc ions, promoting nanoparticle stabilization. Conversely, low-salt conditions may impair enzyme activity and reduce nanoparticle yield. These findings are consistent with previous reports underscoring the importance of ionic strength and medium composition in microbial nanoparticle biosynthesis [51, 73, 74].

Temperature optimization revealed 37 °C as the most effective for both strains, supporting prior studies that identified mesophilic conditions as ideal for microbial nanoparticle biosynthesis [43, 75].

Overall, the results highlight the importance of strain-specific optimization in microbial ZnNP biosynthesis. The distinct responses of S4 and S6 to environmental and chemical conditions underscore the need for tailored approaches, especially when scaling up production or targeting specific nanoparticle properties for applications in medicine, agriculture, or environmental remediation.

Lyophilization was employed for nanoparticle recovery after optimizing the reaction conditions due to its efficiency and convenience. However, it is acknowledged that this method can sometimes affect nanoparticle stability, particularly by promoting aggregation or altering surface properties. These changes may, in turn, influence the physicochemical behavior and biological activity of the ZnNPs [76]. As potential alternatives, spray drying or supercritical fluid drying could be considered in future studies. These techniques may offer gentler dehydration and better control over particle dispersion and morphology, thereby preserving nanoparticle functionality more effectively [19, 77].

The biosynthesis of zinc-based nanoparticles by strains S4 and S6 was confirmed using multiple complementary techniques. Transmission electron microscopy (TEM) revealed distinct morphologies: *A. spanius* (S4) produced spherical ZnS nanoparticles, while *P. resinovorans* (S6) synthesized rod-shaped ZnO–ZnS mixed nanostructures. The particle sizes were consistent with those typically reported for biologically synthesized zinc nanoparticles [52, 78, 79].

Nanoparticle morphology is strongly influenced by synthesis parameters such as pH, precursor concentration, and reaction kinetics. In particular, alkaline conditions, exemplified by the S4 system, favor spherical ZnNP formation due to slower nucleation and more uniform growth [53, 80]. Conversely, acidic environments, as seen with S6, promote anisotropic growth, leading to rod-shaped nanoparticles. This is attributed to rapid nucleation and protonation effects that alter crystal facet stabilization and direct elongation along specific axes [82, 81, 83]. These pH-dependent mechanisms have been widely documented in both biological and chemical synthesis routes, underscoring pH as a critical factor in controlling nanoparticle shape and size [53, 83].

EDX analysis confirmed the elemental purity of the nanoparticles, with major peaks corresponding to Zn and S, and in the case of S6, a combination of Zn, O, and S. These results suggest the formation of either pure ZnS or hybrid ZnO–ZnS structures, likely influenced by the biochemical conditions of the synthesis environment. The presence of additional elements may be attributed to residual components from the culture medium or biomolecules that act as stabilizers [85].

XRD patterns provided definitive insight into the crystalline structure of the nanoparticles. The ZnS nanoparticles from S4 exhibited a cubic structure, as evidenced by distinct diffraction peaks, while the particles synthesized by S6 exhibited a combination of hexagonal ZnO and cubic ZnS phases. This dual-phase structure has been previously observed in mixed-metal oxide nanomaterials [86, 87], suggesting that S6 has the biochemical capability to mediate the formation of complex nanostructures.

FTIR spectroscopy provided additional evidence of the involvement of biological molecules in nanoparticle synthesis and stabilization. Functional groups corresponding to hydroxyl, carboxyl, and amide bonds were prominent in both spectra, indicating the role of proteins and other organic molecules in reducing and capping the nanoparticles [88, 89]. Specifically, the presence of amide II bands and COO⁻ stretches strongly suggests that proteins in the bacterial supernatant facilitated the nucleation and stabilization of the ZnNPs. Characteristic bands in the low wavenumber region (382–618 cm⁻¹) confirm the presence of ZnS and ZnO bonds, further validating the compositional findings from EDX and XRD [91,90, 92].

Overall, the data confirm that both S4 and S6 strains are capable of producing distinct forms of zinc nanoparticles, with differences in morphology, composition, and crystallinity. These differences likely reflect the unique metabolic and enzymatic environments provided by each strain, highlighting their potential for tailored nanoparticle synthesis in various biotechnological applications.

The escalating emergence of antibiotic-resistant pathogens underscores the urgent need for novel antimicrobial agents, particularly those derived from natural or biosynthetic sources [93]. Zinc-based nanoparticles (ZnNPs) have garnered considerable attention due to their potent antimicrobial properties, largely attributed to their nanoscale dimensions, morphology, surface area, and degree of dispersion [94, 95]. Specifically, smaller nanoparticles provide a higher surface-to-volume ratio, enhancing their capacity to disrupt microbial membranes and induce the generation of reactive oxygen species (ROS) such as hydroxyl radicals, superoxide anions, and hydrogen peroxide. These ROS cause extensive damage to microbial membranes, proteins, and DNA, ultimately leading to cell death [96].

In addition to oxidative stress, Zn²⁺ ions released from ZnNPs interfere with critical enzymatic activities and metabolic pathways within microbial cells, further compromising membrane integrity and increasing permeability [53]. Moreover, direct interactions between nanoparticles and microbial membranes result in mechanical damage and destabilization, facilitated by electrostatic attraction between the positively charged nanoparticles and negatively charged bacterial surfaces [97]. Notably, the dual-phase composition of ZnS–ZnO nanocomposites likely amplifies these antimicrobial effects via synergistic oxidative activity and enhanced surface reactivity, while their nanoscale size allows efficient cell wall penetration and intracellular disruption.

From an environmental perspective, biologically synthesized ZnNPs present a safer and more eco-friendly alternative to conventional chemical disinfectants. Their microbial origin and the absence of toxic reducing agents during synthesis contribute to lower ecological toxicity [98, 99]. However, to ensure their safe and effective use in applications such as sludge treatment and environmental remediation, the potential long-term impacts on soil microbiota and aquatic ecosystems must be carefully evaluated. Previous studies have indicated that ZnNPs at moderate concentrations (typically below 100 µg/ml) exhibit minimal cytotoxicity toward non-target organisms while maintaining robust antimicrobial activity [100]. Nevertheless, comprehensive ecotoxicological assessments are necessary to fully understand nanoparticle bioaccumulation, persistence, and degradation in environmental matrices [101].

In this study, biosynthesized ZnS nanoparticles derived from *Achromobacter spanius* (S4) exhibited selective and potent antibacterial activity, particularly against *Staphylococcus epidermidis*. This effectiveness may be linked to their relatively small, spherical morphology, as confirmed by TEM analysis. The ZnS–ZnO hybrid nanoparticles synthesized by *Pseudomonas resinovorans* (S6) demonstrated pronounced activity against *Klebsiella pneumoniae*, a Gram-negative pathogen known for its multiple resistance mechanisms. These observations are consistent with earlier findings highlighting the antimicrobial efficacy of ZnO nanoparticles in managing bacterial skin infections caused by *Staphylococcus aureus* [43].

Importantly, both nanoparticle types surpassed the antimicrobial performance of conventional antibiotics against most tested microbial strains, underscoring their promise as alternative or adjunctive antimicrobial agents. However, the comparatively lower efficacy against *Enterococcus faecalis* suggests that ZnNP antimicrobial mechanisms may vary depending on the structural and metabolic characteristics of different pathogens.

Beyond their antimicrobial potential, this study also evaluated the application of ZnNPs in sludge treatment. The ZnS–ZnO nanoparticles synthesized by strain S6 exhibited significantly higher microbial inhibition compared to the ZnS nanoparticles produced by strain S4, suggesting greater potential for use in wastewater or sludge bioremediation. These findings are consistent with prior research by Vielkind et al. [74], who reported inhibitory effects of ZnO nanoparticles on *Pseudomonas putida* under environmental conditions. Similarly, studies have shown that ZnO and ZnS nanoparticles can reduce the phytotoxicity of sewage sludge by promoting root development in plants [102], and ZnO nanoparticles have been demonstrated to interfere with the anaerobic digestion of waste-activated sludge by inhibiting the reduction of volatile suspended solids [103]. Collectively, these data reinforce the dual functionality of biosynthesized ZnNPs as both effective antimicrobial agents and promising environmental disinfectants for sludge and wastewater treatment applications.

## Conclusion

This study successfully demonstrated the biogenic synthesis of zinc sulfide nanoparticles (ZnS-NPs) and mixed-phase zinc sulfide-oxide nanoparticles (ZnS-ZnO NPs) using two distinct bacterial strains: *Achromobacter* sp. S4 and *Pseudomonas* sp. S6. The biosynthesis process was visually indicated by a color change in the cell supernatants. Characterization via TEM, EDX, XRD, and FT-IR revealed that *Achromobacter*-derived ZnS NPs were spherical (14.1–29 nm), while *Pseudomonas*-derived ZnS-ZnO NPs exhibited a rod-shaped morphology (3.93–43.9 nm).

Functionally, ZnS-NPs from S4 showed antimicrobial activity against *Klebsiella pneumoniae*, while ZnS-ZnO NPs from S6 were more effective against *Staphylococcus epidermidis*. In sludge treatment assays, ZnS-ZnO NPs (25 µg/ml) demonstrated superior performance in reducing microbial load compared to single-phase ZnS-NPs. This study highlights the potential of eco-friendly, cost-effective ZnNPs for combating pathogenic bacteria and remediating sludge.

### Limitations, strengths, and future directions

This study highlights the potential of eco-friendly, biosynthesized ZnNPs as cost-effective agents for antimicrobial applications and environmental remediation. Key strengths include the use of green synthesis pathways, strain-specific optimization, and comprehensive physicochemical characterization. Nonetheless, limitations exist, such as the narrow scope of biological activity assessment and the absence of in vivo testing. Future research should explore a broader range of biological effects—such as antioxidant, anti-inflammatory, and catalytic activities—and conduct real-world application studies. Importantly, the scalability of this biosynthetic approach also warrants further investigation to support industrial production and large-scale environmental applications.

## Data Availability

Sequence data that support the findings of this study have been deposited in the European Nucleotide Archive with the primary accession codes PP593876.1 and PP535560.1.
